# Correction: Evaluating immune response and metabolic related biomarkers pre-allogenic hematopoietic stem cell transplant in acute myeloid leukemia

**DOI:** 10.1371/journal.pone.0313701

**Published:** 2024-11-07

**Authors:** The PLOS ONE Editors

## Notice of republication

This article was republished on October 31, 2024, to address an issue identified post-publication and provide an updated version of S1 File. Please download this article again to view the correct version.

## References

[1] Siamakpour-Reihani S, Cao F, Lyu J, Ren Y, Nixon AB, Xie J, et al. (2022) Evaluating immune response and metabolic related biomarkers pre-allogenic hematopoietic stem cell transplant in acute myeloid leukemia. PLoS ONE 17(6): e0268963. 10.1371/journal.pone.0268963 35700185 PMC9197059

