# Supplementary material for: Evaluating immune response and metabolic related biomarkers pre-allogenic hematopoietic stem cell transplant in acute myeloid leukemia
Source: PLoS One. 2022 Jun 14;17(6):e0268963. doi: 10.1371/journal.pone.0268963 (PMC9197059; doi:10.1371/journal.pone.0268963)

Analysis 1

- Aim 1: To determine the association between Metablomics biomarker levels and age groups (younger than 30 vs older than 55) in HCT (hematopoietic stem cell transplant) patients in baseline samples.

Wilcoxon Rank Sum Test Function

Perform Wilcoxon rank sum test to compare biomarker levels of baseline in younger group and older group.

```
analysis1 <- function(data, marker, label) {
    p_val = c()
    for (i in 1:length(marker)) {
        testr <- wilcox.test(data[, marker[i]] ~ `Age Grp`, data = data,
            alternative = "two.sided", paired = F)
        p_val <- c(p_val, testr$p.value)
    }
    q_val <- p.adjust(p_val, method = "fdr")
    comosum1 <- data.frame(marker, p_val, q_val) %>% rename(`:=`(!!label,
        "marker")) %>% arrange(p_val)
    return(comosum1)
}
```

Summary for Comparision

Markers	p_val	q_val
NEFA	0.17082	0.84573
Glycerol	0.46265	0.84573
HBUT	0.65261	0.84573
LACT	0.67659	0.84573
TG-B	0.93671	0.93671

Q-values are from FDR approach for multiplicity.

Summary for Comparision

Amino Acids Markers	p_val	q_val
Arg	0.08826	0.58308
Phe	0.15146	0.58308
His	0.23002	0.58308
Orn	0.30105	0.58308
Glx	0.31680	0.58308
Ser	0.33307	0.58308
Asx	0.33307	0.58308
Met	0.34985	0.58308
Cit	0.34985	0.58308
Val	0.44135	0.66202
Tyr	0.52324	0.70627
Ala	0.61210	0.70627
Pro	0.61210	0.70627
Leu/Ile	0.75613	0.81014
Gly	0.98696	0.98696

Q-values are from FDR approach for multiplicity.

Summary for Comparision

Acyl Carnitines Markers	p_val	q_val
C18:2-OH	0.00155	0.03132
C4-DC/Ci4-DC	0.00219	0.03132
C8:1-OH/C6:1-DC	0.00369	0.03132
C12-OH/C10-DC	0.00375	0.03132
C20-OH/C18-DC	0.00415	0.03132
C14:1-OH	0.00418	0.03132
C16:1	0.00491	0.03156
C10-OH/C8-DC	0.00599	0.03367
C18-OH/C16-DC	0.00791	0.03954
C14-OH/C12-DC	0.01320	0.05088
C8:1-DC	0.01357	0.05088
C14:1	0.01357	0.05088
C8:1	0.01817	0.06290
C14:2	0.02136	0.06866
C16-OH/C14-DC	0.02871	0.08093
C8	0.02878	0.08093
C10:3	0.03848	0.10014
C18:2	0.04006	0.10014
C18:1-DC	0.04329	0.10253
C18:1-OH/C16:1-DC	0.04676	0.10520
C4-OH	0.05038	0.10797
C18:1	0.06075	0.11904
C14	0.06127	0.11904
C10:2	0.06349	0.11904
C10:1	0.07924	0.14178

Q-values are from FDR approach for multiplicity.

Summary for Comparision

Acyl Carnitines Markers	p_val	q_val
C16:2	0.08248	0.14178
C6-DC/C8-OH	0.08507	0.14178
C10	0.09122	0.14660
C20:4	0.09549	0.14817
C16:1-OH/C14:1-DC	0.11153	0.16730
C2	0.16126	0.23357
C7-DC	0.16610	0.23357
C16	0.19347	0.26382
C18	0.34521	0.44980
C12:1	0.34985	0.44980
C4/Ci4	0.36529	0.45153
C5-DC	0.37126	0.45153
C6	0.47838	0.56651
C5:1	0.50208	0.57933
C5	0.57332	0.64499
C12	0.63529	0.69727
C22	0.70540	0.75578
C20	0.79846	0.83559
C5-OH/C3-DC	0.93401	0.95524
C3	1.00000	1.00000

Q-values are from FDR approach for multiplicity.

Summary for Comparision

Median and range details of significant markers in each age group.

Biomarker	Median(Range) of Younger	Median(Range) of Older
C18:2-OH	0.005 (0.001 - 0.01)	0.007 (0.004 - 0.012)
C4-DC/Ci4-DC	0.026 (0.01 - 0.036)	0.031 (0.014 - 0.08)
C8:1-OH/C6:1-DC	0.022 (0.011 - 0.038)	0.032 (0.018 - 0.082)
C12-OH/C10-DC	0.004 (0.002 - 0.013)	0.008 (0.005 - 0.024)
C20-OH/C18-DC	0.006 (0.003 - 0.011)	0.009 (0.004 - 0.019)
C14:1-OH	0.009 (0.005 - 0.018)	0.015 (0.006 - 0.031)
C16:1	0.016 (0.004 - 0.045)	0.031 (0.011 - 0.116)
C10-OH/C8-DC	0.025 (0.007 - 0.065)	0.043 (0.019 - 0.071)
C18-OH/C16-DC	0.005 (0.002 - 0.01)	0.007 (0.002 - 0.014)

Older patients tend to have higher values of these markers.

Boxplots of Biomarker Levels in Different Age Groups

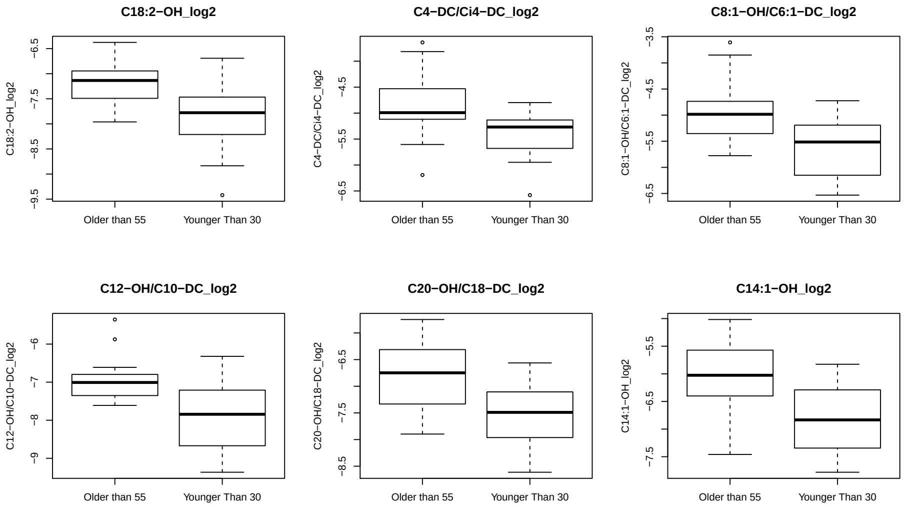

Boxplots of Biomarker Levels in Different Age Groups

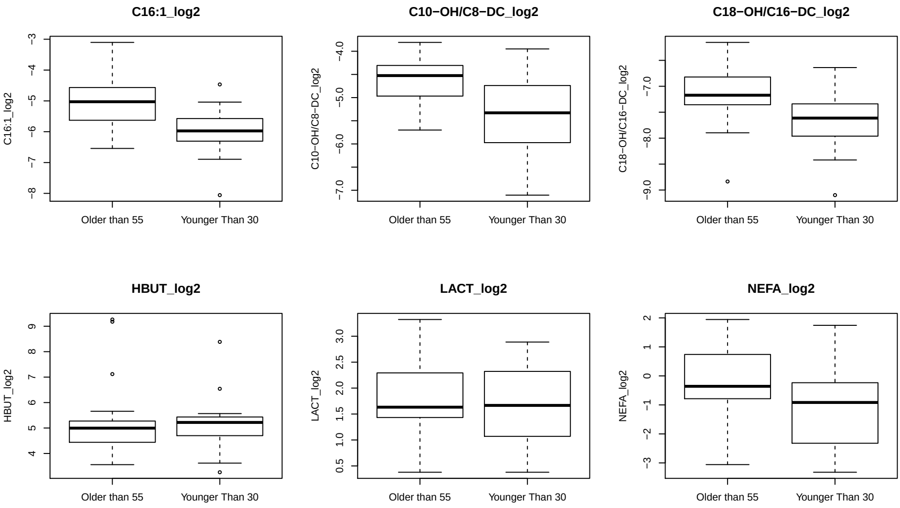

Boxplots of Biomarker Levels in Different Age Groups

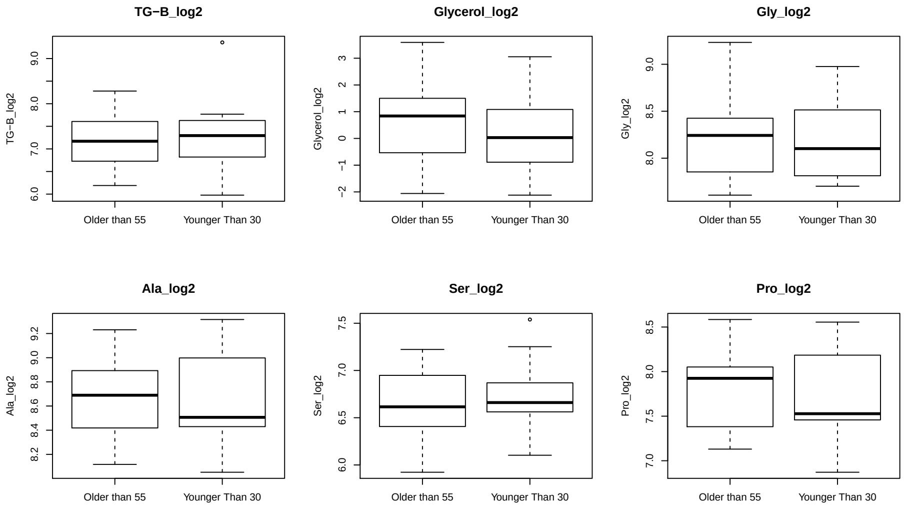

Boxplots of Biomarker Levels in Different Age Groups

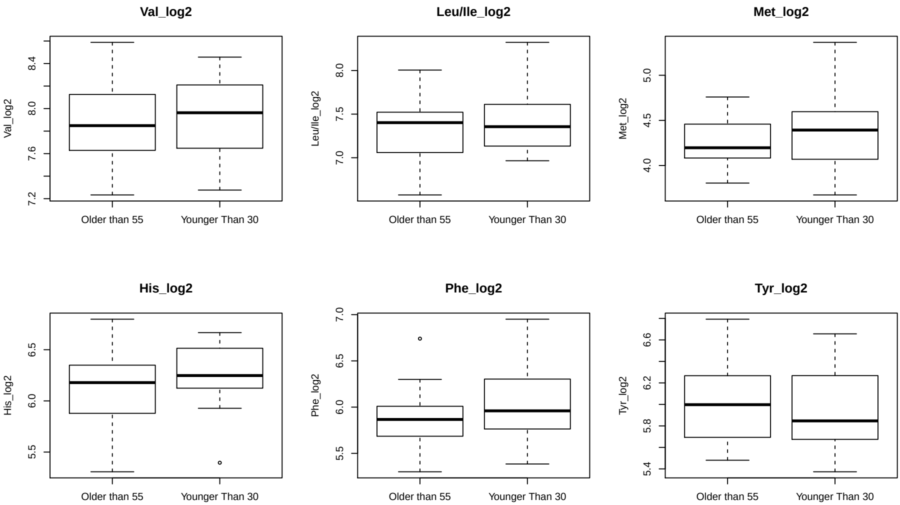

Boxplots of Biomarker Levels in Different Age Groups

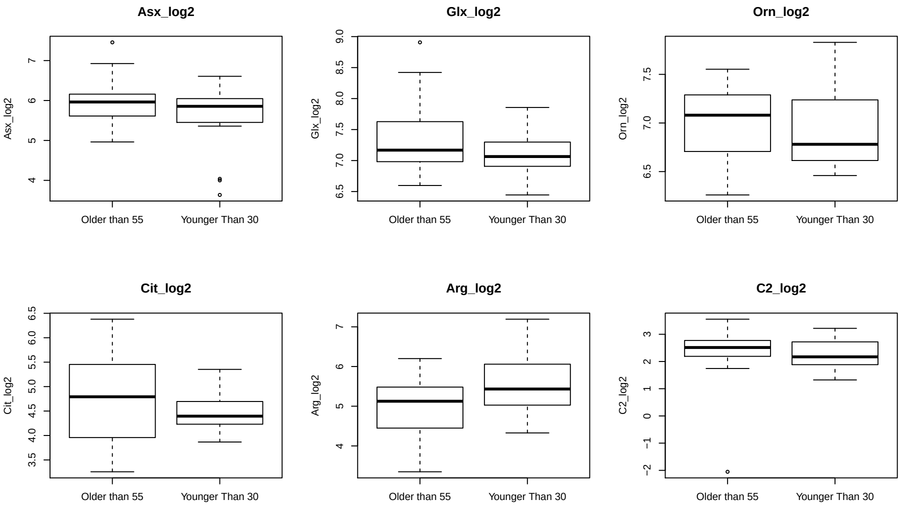

Boxplots of Biomarker Levels in Different Age Groups

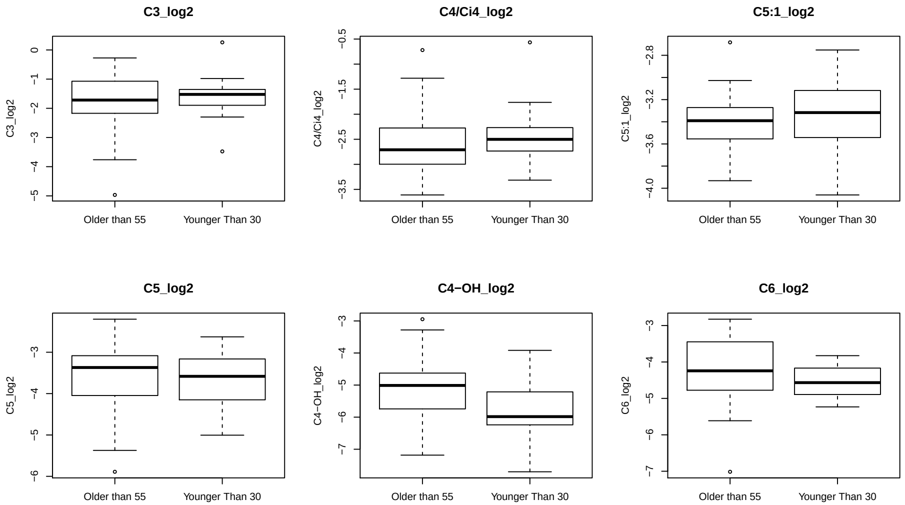

Boxplots of Biomarker Levels in Different Age Groups

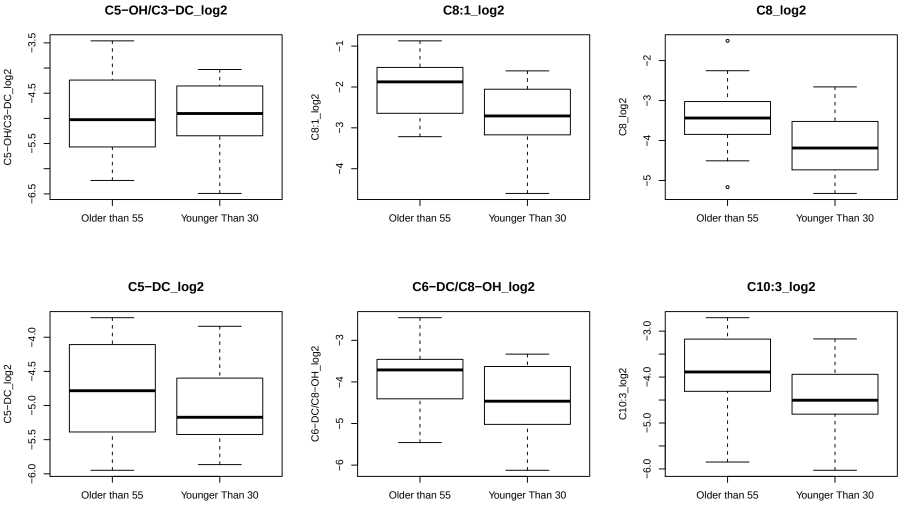

Boxplots of Biomarker Levels in Different Age Groups

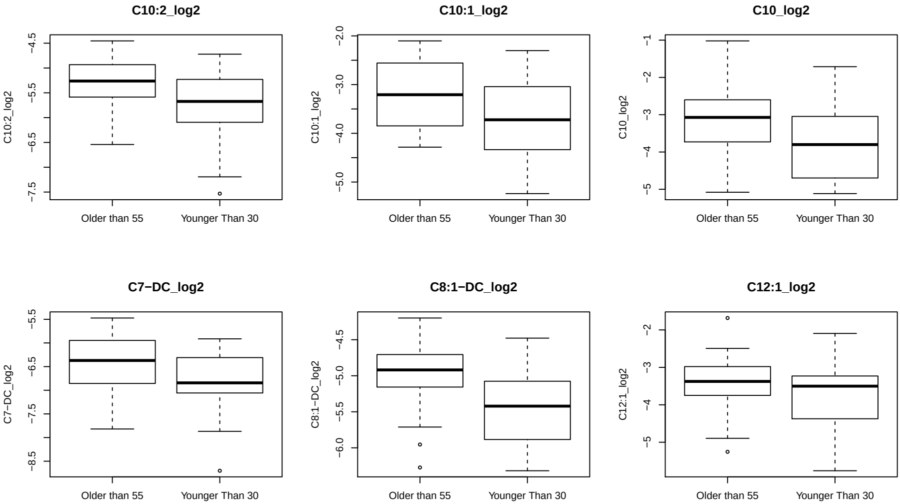

Boxplots of Biomarker Levels in Different Age Groups

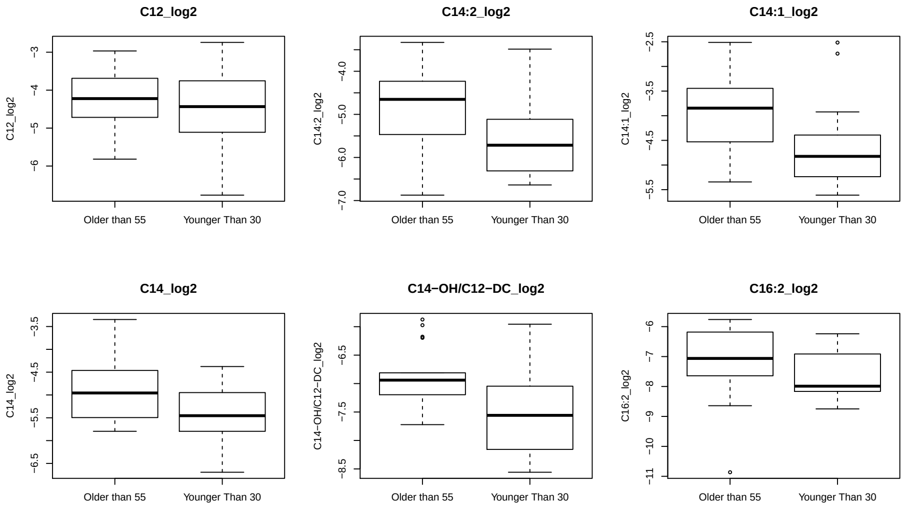

Boxplots of Biomarker Levels in Different Age Groups

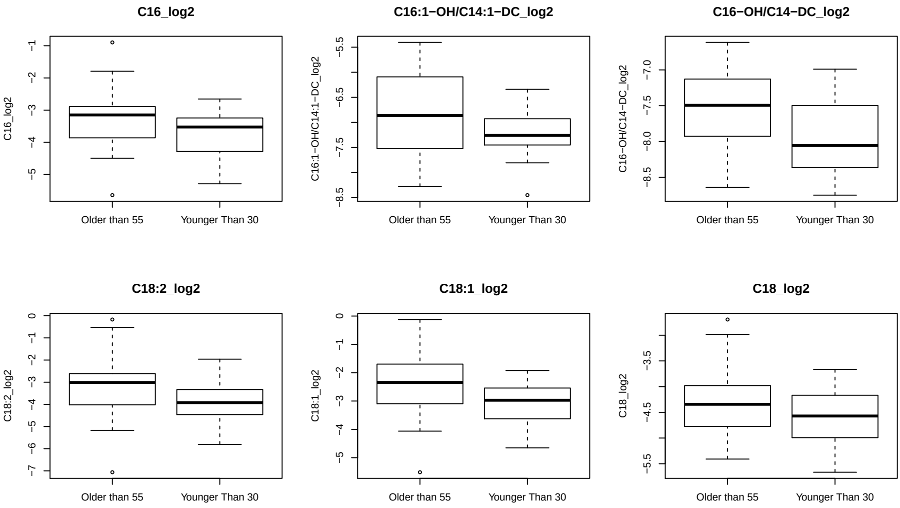

Boxplots of Biomarker Levels in Different Age Groups

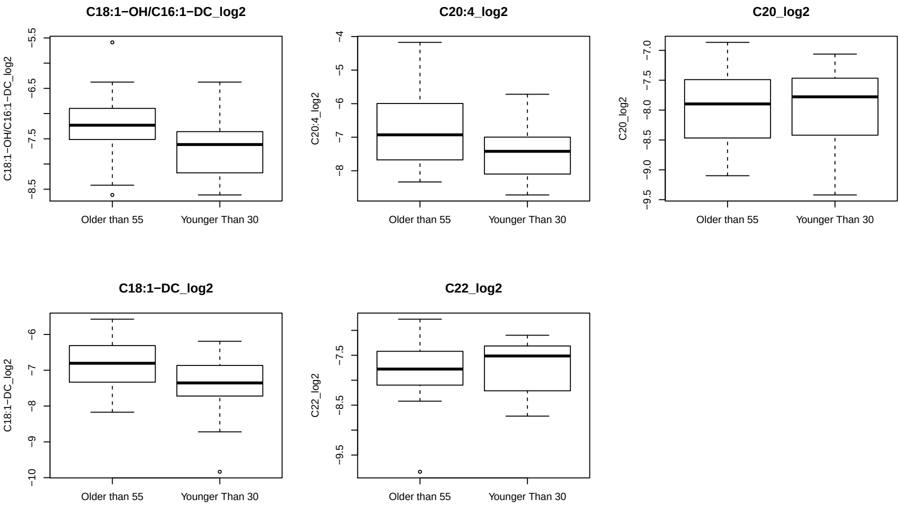

Supplement: S2 File — (PDF) [file pone.0268963.s002.pdf]
