# Supplementary material for: Evaluating immune response and metabolic related biomarkers pre-allogenic hematopoietic stem cell transplant in acute myeloid leukemia
Source: PLoS One. 2022 Jun 14;17(6):e0268963. doi: 10.1371/journal.pone.0268963 (PMC9197059; doi:10.1371/journal.pone.0268963)
Supplement: Attachment — Submitted filename: Response to Reviewers-Reihani- Sung etal -03-08-2022.docx [file pone.0268963.s003.docx]

PONE-D-21-39481

03/04/2022

**Rebuttal letter**

**We are greatly appreciative for this careful and detailed review. Please see a point-by-point response to the reviewers’ comments. We have revised the paper accordingly.**

Journal Requirements:

When submitting your revision, we need you to address these additional requirements.

1. Please ensure that your manuscript meets PLOS ONE's style requirements, including those for file naming. The PLOS ONE style templates can be found at
   <https://journals.plos.org/plosone/s/file?id=wjVg/PLOSOne_formatting_sample_main_body.pdf>
2. and
   <https://journals.plos.org/plosone/s/file?id=ba62/PLOSOne_formatting_sample_title_authors_affiliations.pdf>

2. Please provide additional details regarding participant consent. In the ethics statement in the Methods and online submission information, please ensure that you have specified (1) whether consent was informed and (2) what type you obtained (for instance, written or verbal, and if verbal, how it was documented and witnessed). If your study included minors, state whether you obtained consent from parents or guardians. If the need for consent was waived by the ethics committee, please include this information.

If you are reporting a retrospective study of medical records or archived samples, please ensure that you have discussed whether all data were fully anonymized before you accessed them and/or whether the IRB or ethics committee waived the requirement for informed consent. If patients provided informed written consent to have data from their medical records used in research, please include this information

Once you have amended this/these statement(s) in the Methods section of the manuscript, please add the same text to the “Ethics Statement” field of the submission form (via “Edit Submission”).

For additional information about PLOS ONE ethical requirements for human subjects research, please refer to <http://journals.plos.org/plosone/s/submission-guidelines#loc-human-subjects-research>.

**Response:** Written, witnessed informed consent was obtained for sample collection for future research purposes, as well as use of associated clinical data. De-identified samples and de-identified clinical data were accessed under a separate, IRB-approved protocol; because deidentified samples and data were used, and patients already consented to future use of samples for research purposes, the IRB waived the requirement for consent for this use protocol. (IRB #s are: Pro00006268 and Pro00100650).:

3. Thank you for stating the following in the Acknowledgments Section of your manuscript:

"This research was in part supported, in part, by the American Society of Hematology (ASH) Scholar Award and the NIH/National Institute on Aging 1R21AG066388-01 award"

We note that you have provided funding information that is not currently declared in your Funding Statement. However, funding information should not appear in the Acknowledgments section or other areas of your manuscript. We will only publish funding information present in the Funding Statement section of the online submission form. 
Please remove any funding-related text from the manuscript and let us know how you would like to update your Funding Statement. Currently, your Funding Statement reads as follows:

"YES - Specify the role(s) played
A.D.S.:
American Society of Hematology (ASH) 
https://www.hematology.org/
Scholar Award and the NIH/National Institute on Aging 1R21AG066388-01 award.
https://reporter.nih.gov/search/5NyHmfz0skuWJC2JAuThRw/project-details/9980757"

Please include your amended statements within your cover letter; we will change the online submission form on your behalf.

**Response:** We have removed funding-related text from the manuscript. **In the cover letter** we have requested the following funding information (* is a new request):

We also would like to update our Funding Statement to include the following information (per your request this section has bene removed from the acknowledge section):

1. **American Society of Hematology (ASH),** **(PI: Anthony D Sung)**

<https://www.hematology.org/>

2. **NIH/National Institute on Aging 1R21AG066388-01 award (PI: Anthony D Sung).**

<https://reporter.nih.gov/search/5NyHmfz0skuWJC2JAuThRw/project-details/9980757>

3. **NIH/National Institute on Aging Duke Pepper Older Americans Independence Center**

**P30 AG028716, (PI: Schmader, Mini #6, PI of Mini: Anthony D Sung)**

<https://reporter.nih.gov/search/KsvVX_8rwUKk6CXVf2k0vg/project-details/9971412>

4. In your Data Availability statement, you have not specified where the minimal data set underlying the results described in your manuscript can be found. PLOS defines a study's minimal data set as the underlying data used to reach the conclusions drawn in the manuscript and any additional data required to replicate the reported study findings in their entirety. All PLOS journals require that the minimal data set be made fully available. For more information about our data policy, please see http://journals.plos.org/plosone/s/data-availability.

"Upon re-submitting your revised manuscript, please upload your study’s minimal underlying data set as either Supporting Information files or to a stable, public repository and include the relevant URLs, DOIs, or accession numbers within your revised cover letter. For a list of acceptable repositories, please see http://journals.plos.org/plosone/s/data-availability#loc-recommended-repositories. Any potentially identifying patient information must be fully anonymized.

Important: If there are ethical or legal restrictions to sharing your data publicly, please explain these restrictions in detail. Please see our guidelines for more information on what we consider unacceptable restrictions to publicly sharing data: http://journals.plos.org/plosone/s/data-availability#loc-unacceptable-data-access-restrictions. Note that it is not acceptable for the authors to be the sole named individuals responsible for ensuring data access.

We will update your Data Availability statement to reflect the information you provide in your cover letter.

**Response:** The minimal data set underlying the results described in our manuscript have been uploaded as supplement/ supporting document. We have also added this statement to the cover letter: “This revised manuscript includes the revisions and edits requested by reviewers. In addition, for the re-submission of our revised manuscript, we have uploaded our study’s minimal underlying data set as Supporting Information files.”

5. Please include captions for your Supporting Information files at the end of your manuscript, and update any in-text citations to match accordingly. Please see our Supporting Information guidelines for more information: http://journals.plos.org/plosone/s/supporting-information.

[Note: HTML markup is below. Please do not edit.]

Reviewers' comments:

Reviewer's Responses to Questions

**Comments to the Author**

**Reviewer #1: Comments:**
1. Overall survival (OS) is spelled out twice in the abstract.

**Response: This has been corrected**

2. Abstract Line 32: “This retrospective study was designed with an exploratory and hypothesis generating….” Introduction Line 113-114, “We hypothesized that metabolomics and blood-based biomarkers have the potential to be used as predictive biomarkers for AML patients who receive HCT treatment.” Please clearly state the hypothesis within the abstract.

**Response**: Revisions have been added

3. Please spell out all abbreviations upon first usage. For example, MMP-3 is spelled out on line 152, but first used on line 99.

**Response:** Revisions have been added

4. Methods: For biomarkers of inflammation and aging, please provide more details regarding how the 54-plex plates were read. What equipment did you use? Were serial dilutions performed?

**Response:** Revisions have been added to the methods sub- section for “Biomarkers of inflammation and aging”

5. The metabolomics data are presented haphazardly in the Results section. They are hard to follow. Revision is recommended.

**Response:** The section has been revised in results section.

6. Discussion: A recent article (PMID: 34722316) regarding BET inhibition in GVHD after HCT should be discussed in relation to IL-23/IL-17.

**Response:** This has been added to the discussion section

7. Discussion Lines 341-346 are not necessary and do not have relevance to the current manuscript.

**Response:** Section removed

8. Do the authors speculate that increased acylcarnitines in older patients correlates with enhanced oxidative phosphorylation? In what cell type? This would be supported by the lower mean levels of lactate. Please clarify within the text.

**Response:** We show no significant difference with age for lactate, so would not appear to support an association with the increase in long/median-chain AC with age. Our data do however show a significant decrease in lactate with aGVHD, as they also do decreases in various ACs. This could potentially signify a pathway emphasis towards Krebs (and oxidative phosphorylation) and away from lactate or fatty acid synthesis, or alternatively a drive in the opposite direction through pyruvate towards gluconeogenesis. In order to have the data and conclusion, we would need a more intensive study to support such claims (this would be out of the scope for this paper).

A paragraph also has been added with references to the discussion related to this comment.

9. Figure 1, panel labels are recommended (e.g. A, B, C, D). These should be updated within the text. Please use consistent font and font sizes for all graph labels.

**Response:** The figure has been revised and updated within the text

10. Figure legends: Please indicate the meaning of * within the figures.

**Response:** Please note there is no * sign in the figures. The only * signs are for table 2 with the explanation of: “* are separate ELISAs not part of the 54-plex. ** Higher baseline levels in younger patients”.

However, in the figures there are dots/points (which might have been mistaken for *), so we have added the following to Fig 1 legend: data is presented as a boxplot. Boxes represent 25th (Q1) and 75th (Q3) percentiles; Horizontal lines indicate the medians; Upper whiskers indicate maximum; Lower whiskers indicate minimum. Points indicate any observations outside the whiskers.”

11. There is a lot of supporting information that is not described or called out within the manuscript text.

**Response:** We have added more data and supporting information, so we hope we have responded to the comment to an extent (since the comment is general, we did not know if adding specific information was intended).

Reviewer #2: Siamakpour-Reihani et al investigate the expression of a large number of immune/inflammatory and metabolomic biomarkers in plasma samples from patients with acute myeloid leukemia (AML) before hematopoietic stem cell transplantation (HCT) and explore associations with disease outcome (overall survival-OS), young (≤30years) or old age (≥ 60 years) and other clinical outcomes (chronic graft versus host disease-cGVHD, acute graft versus host disease-aGVHD, infection and relapse). They found that several biomarkers were elevated in older patients compared to younger patients, whereas only IL23 was upregulated in younger patients. Three markers (IL13, SAA, CRP) were lower in patients with OS ≥ 3 years, whereas IL-9, Eotaxin-3 was associated with aGVHD, Flt-1 with cGVHD, D-dimer with infection, or IL-17D, bFGF, and Eotaxin-3 with relapse. Higher baseline levels of medium- and long-chain acylcarnitines (AC) were found in older patients, whereas lactate and long-chain AC were associated with aGVHD, and medium-chain AC with cGVHD. These findings are interesting, and the manuscript is well-presented; however, the following issues need attention.
1. Sex and age matched healthy controls are missing. Thus, it is not clear whether the differential expression of immune/inflammatory and metabolomic biomarkers in plasma samples from AML patients owes to disease, age or comorbidities related to elder.

**Response:** lack of matched healthy controls is a limitation of our study, we have add this to our “limitation section of the manuscript”. In this retrospective study the comparison is only made between the blood based biomarkers in different groups of AML patients based on age and survival. Also, we did not have access to matched healthy control samples for this retrospective study.

2. The characteristics of the patients in Table 1, should be presented in more detail. All the parameters examined (cGVHD, aGVHD, infection, relapse, deaths, etc), as well as disease comorbidities (such as thrombosis, cardiac disease, etc) must be presented.

3. In this context, it should be excluded that the factors that are differentially expressed in the various subsets of AML patients, do not owe their differential expression to an infection or comorbidity.

**Response (#2):** Table 1 have been updated to present the parameters and conditions examined.

**Response (#3):** Based on the updated Table 1, we have not seen balance issue between age groups in our outcomes. The p-values are not significant for differences between groups for infection or other comorbidities we studied.

The only two significant p-values for difference between the two groups in Table 1 are: for the age (which was by design), and for Conditioning (which is expected since the conditioning for younger HCT patients will be different

4. Metabolomic analysis was performed in retrospectively collected samples. Were the samples collected appropriately for this kind of analysis? The samples were collected at similar period of the day, were the patients fasting? All this information needs to be included in the manuscript (if they were not performed, along with comment #1, they should be discussed).

**Response:** All samples were appropriate for the metabolomics assays as the DPMI core is highly specialized in these studies.

The blood samples have been collected as part of the ABMT Biorepository protocol and we used them retrospectively. However, the samples are not collected at the same time of the day. As part of our IRB approved protocol the biorepository samples have been collected during routine appointments pre and post HCT at routine lab visits. Thus, they are not all collected at the same time. The patients are not required to be fasting unless requested by the physician.

5. It is not reported why the authors chose these age limits (e.g., why young were considered those under 30 years and not those under 32 or 35 years). It was based on statistical analysis, or it was an arbitrary selection? This must be clearly stated.

**Response:** The information was added to the methods section: The age cut-offs were arbitrary selection and mostly based on what we had samples for. Though there is support that AML in patients >55years behaves differently than AML in patients <55 years (Appelbaum, Gundacker et al. 2006).

Appelbaum, F. R., H. Gundacker, D. R. Head, M. L. Slovak, C. L. Willman, J. E. Godwin, J. E. Anderson and S. H. Petersdorf (2006). "Age and acute myeloid leukemia." Blood **107**(9): 3481-3485.
