# Supplementary material for: Evaluating immune response and metabolic related biomarkers pre-allogenic hematopoietic stem cell transplant in acute myeloid leukemia
Source: PLoS One. 2022 Jun 14;17(6):e0268963. doi: 10.1371/journal.pone.0268963 (PMC9197059; doi:10.1371/journal.pone.0268963)
Supplement: Attachment — Submitted filename: Response to Reviewers_ Reihani_Sung et al 04-11-22.docx [file pone.0268963.s004.docx]

04-11-2022

**Rebuttal letter:**

**Thank you for your time and comments. We are greatly appreciative for this second review on the revisions previously made. Please see the response to the additional comments and minor changes requested (blue color font). We have revised the paper accordingly.**

PONE-D-21-39481R1

Evaluating immune response and metabolic related biomarkers pre-allogenic hematopoietic stem cell transplant in acute myeloid leukemia

PLOS ONE

Please include the following items when submitting your revised manuscript:

- A rebuttal letter that responds to each point raised by the academic editor and reviewer(s). You should upload this letter as a separate file labeled 'Response to Reviewers'.

**Response:** This had been done

- A marked-up copy of your manuscript that highlights changes made to the original version. You should upload this as a separate file labeled 'Revised Manuscript with Track Changes'.

**Response:** This had been done

- An unmarked version of your revised paper without tracked changes. You should upload this as a separate file labeled 'Manuscript'.
- Response: This had been done

Reviewers' comments:

Reviewer's Responses to Questions

**Comments to the Author**

1. If the authors have adequately addressed your comments raised in a previous round of review and you feel that this manuscript is now acceptable for publication, you may indicate that here to bypass the “Comments to the Author” section, enter your conflict of interest statement in the “Confidential to Editor” section, and submit your "Accept" recommendation.

Reviewer #1: All comments have been addressed

Reviewer #2: All comments have been addressed

2. Is the manuscript technically sound, and do the data support the conclusions?

The manuscript must describe a technically sound piece of scientific research with data that supports the conclusions. Experiments must have been conducted rigorously, with appropriate controls, replication, and sample sizes. The conclusions must be drawn appropriately based on the data presented.

Reviewer #1: Yes

Reviewer #2: Yes

3. Has the statistical analysis been performed appropriately and rigorously?

Reviewer #1: No

Reviewer #2: Yes

4. Have the authors made all data underlying the findings in their manuscript fully available?

The [PLOS Data policy](http://www.plosone.org/static/policies.action#sharing) requires authors to make all data underlying the findings described in their manuscript fully available without restriction, with rare exception (please refer to the Data Availability Statement in the manuscript PDF file). The data should be provided as part of the manuscript or its supporting information, or deposited to a public repository. For example, in addition to summary statistics, the data points behind means, medians and variance measures should be available. If there are restrictions on publicly sharing data—e.g. participant privacy or use of data from a third party—those must be specified.

Reviewer #1: Yes

Reviewer #2: Yes

5. Is the manuscript presented in an intelligible fashion and written in standard English?

PLOS ONE does not copyedit accepted manuscripts, so the language in submitted articles must be clear, correct, and unambiguous. Any typographical or grammatical errors should be corrected at revision, so please note any specific errors here.

Reviewer #1: Yes

Reviewer #2: Yes

6. Review Comments to the Author

Please use the space provided to explain your answers to the questions above. You may also include additional comments for the author, including concerns about dual publication, research ethics, or publication ethics. (Please upload your review as an attachment if it exceeds 20,000 characters)

Reviewer #1: Summary: In the present study, the authors evaluated immune/inflammatory responses and metabolic biomarkers associated with overall survival (OS) in acute myeloid leukemia (AML) after hematopoietic stem cell transplantation (HCT). Using plasma from 34 AML patients after HCT, they analyzed 65 blood-based metabolomics markers and 61 immune/inflammatory related biomarkers, comparing long-term OS (>1 year) with short-term OS (<3 years), or comparing younger (<30 years) with older (>55 years) patients. They found several markers that were elevated or reduced in older versus younger patients, as well as many immune/inflammatory markers associated with outcomes such as graft-versus-host disease (GVHD), infection, or relapse. For metabolic markers, they found higher levels of medium- and long-chain acylcarnitines in older patients with GVHD. Further studies would be required to assess the use of these biomarkers in disease prognosis and therapy. Comments for improvement are listed below:

**Comments:**
1. Please provide statistics within each panel of every figure.

**Response:** Thank you for your suggestion. We have added information requested to the figures. Figures 1-3 have been revised. Some additional statistical data has also been added to the figure legends and the main text in the revised manuscript.

2. Supplemental data are still not adequately called out within the text.

**Response:** Information has been added to the manuscript (Lanes 198 and 281)

Reviewer #2: The authors addressed all issues and the manuscript is appropriate for publication.
The only correction that is needed in the new version is the deletion of the phrase in lines 350-351 because it is a repetition of the next phrase.

**Response:** The phrase has been deleted / revised
